# Deep echocardiography: data-efficient supervised and semi-supervised deep learning towards automated diagnosis of cardiac disease

**DOI:** 10.1038/s41746-018-0065-x

**Published:** 2018-10-18

**Authors:** Ali Madani, Jia Rui Ong, Anshul Tibrewal, Mohammad R. K. Mofrad

**Affiliations:** 1 Molecular Cell Biomechanics Laboratory, Departments of Bioengineering and Mechanical Engineering, University of California, Berkeley, California USA; 2 Department of Electrical Engineering and Computer Science, University of California, Berkeley, California USA; 3 UCSF-Berkeley Graduate Program in Bioengineering, Berkeley, California USA

**Keywords:** Heart failure, Echocardiography

## Abstract

Deep learning and computer vision algorithms can deliver highly accurate and automated interpretation of medical imaging to augment and assist clinicians. However, medical imaging presents uniquely pertinent obstacles such as a lack of accessible data or a high-cost of annotation. To address this, we developed data-efficient deep learning classifiers for prediction tasks in cardiology. Using pipeline supervised models to focus relevant structures, we achieve an accuracy of 94.4% for 15-view still-image echocardiographic view classification and 91.2% accuracy for binary left ventricular hypertrophy classification. We then develop semi-supervised generative adversarial network models that can learn from both labeled and unlabeled data in a generalizable fashion. We achieve greater than 80% accuracy in view classification with only 4% of labeled data used in solely supervised techniques and achieve 92.3% accuracy for left ventricular hypertrophy classification. In exploring trade-offs between model type, resolution, data resources, and performance, we present a comprehensive analysis and improvements of efficient deep learning solutions for medical imaging assessment especially in cardiology.

## Introduction

With the improved quality and accessibility of both medical imaging equipment and effective healthcare policy, medical imaging has become an increasingly critical step in modern healthcare diagnostics and procedures. Interpretation of medical imagery requires specialized training and is a time-intensive process. Machine learning and computer vision techniques provide an avenue to augment insights, improve accuracy, and optimize workload time for interpretation. Traditional machine learning techniques in medical imaging involve matching of features hand-engineered by domain experts, a laborious process with limited scope and effectiveness.^1,2^ Recent advances in deep learning,^3–5^ a data-driven approach, and the increasing accessibility of powerful graphical processing units (GPUs)^6,7^ have made the automation of image-based diagnosis insights possible. Researchers have succeeded in applying deep learning techniques in radiology, cardiology, and dermatology,^8–10^ including detecting pneumonia from chest X-rays^11^ and classifying images of benign versus malignant skin lesions.^12^

While deep learning holds great promise in automating the task of medical diagnosis, there remains a set of unique challenges to be resolved before it can be deployed in practice at scale.^8^ Specifically, deep learning algorithms require massive amounts of labeled data to achieve human-level classification performance. Due to privacy laws, differing standards across the healthcare industry and the lack of medical system integration, medical data is less available compared to other fields of computer vision. In addition, medical datasets suffer from class imbalances as certain conditions occur much less frequently than others. Labeling of medical images is complex and requires the time of medical professionals, making it significantly more expensive compared to other computer vision tasks. In addition, there is a limit to the effectiveness of current algorithms in processing high-resolution medical images. As a result, there is a need to identify an optimal balance between resolution size and computational burden. Lastly, medical images often contain other metadata that may be irrelevant for the classification task, leading to less than optimal performance from deep learning models that are unable to filter out this extra information.^13^

In this paper, we address the above challenges by developing data-efficient training methodologies in the domain of transthoracic echocardiograms (TTEs) classification, the most ubiquitous, versatile, and cost-effective cardiac imaging modality available.^14^ Currently, studies have shown surprising rates of echocardiographic assessment inaccuracy can be up to 30% of echo reports^15^ and echocardiographic quality inadequacy in 24% of imaging studies.^16^ Automating interpretation of echocardiograms with trained deep learning classifiers can significantly lower cost, improve quality, and augment cardiologists in making faster and more accurate diagnoses. TTE consists of video clips, still images, and doppler measurements recorded from over a dozen different viewing orientations.^17^ Determining the view is the essential initial step in interpreting an echocardiogram and establishing quality of recorded medical imaging. This step is challenging due to subtle inter-view differences and the variability within a single view.^13^ In addition, from different view orientations further symptoms can be visually identified, such as the enlargement of chambers and thickening of ventricular walls before final prognosis. Left ventricular hypertrophy (LVH), a condition commonly associated with heart disease and hypertension, can be identified by the thickness and relative size of the left ventricle from echocardiograms.^18^

**Fig. 1:**
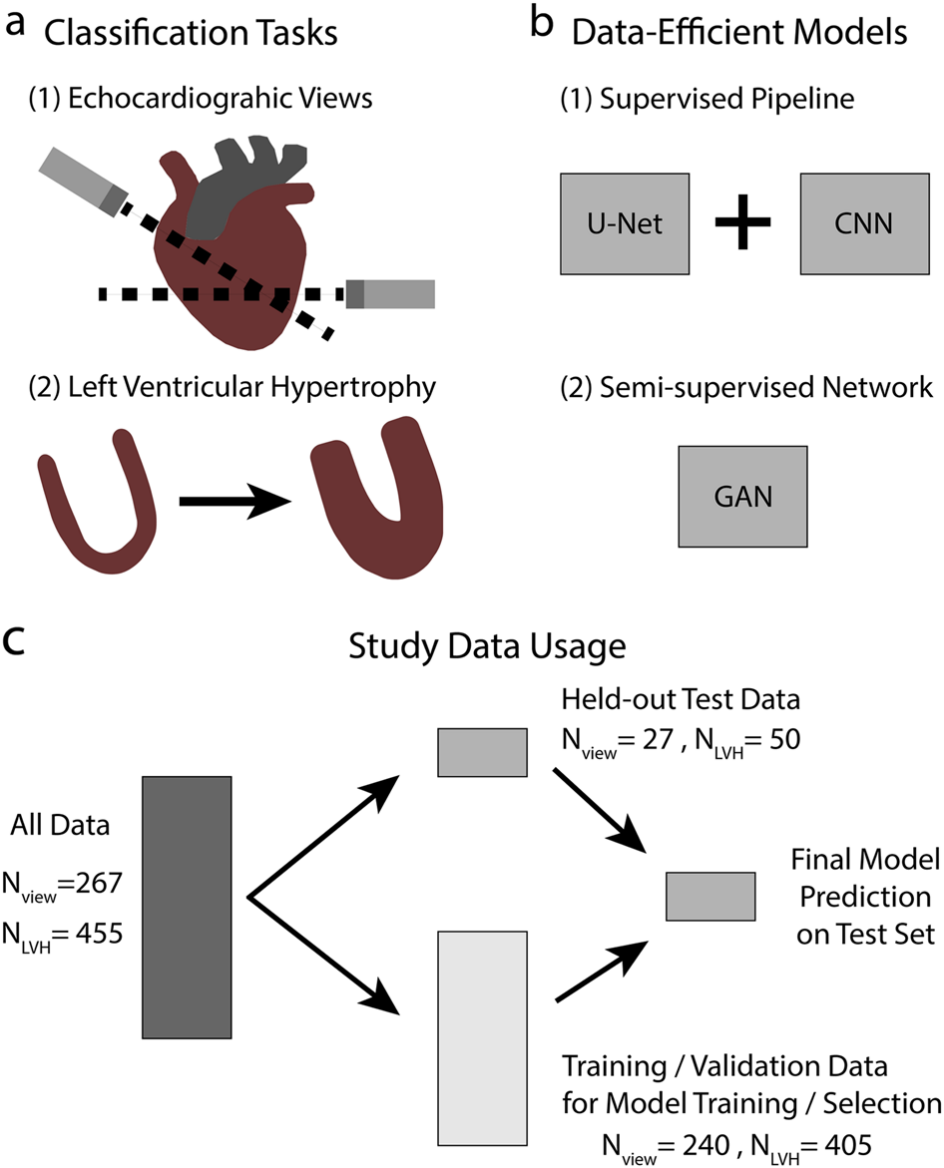
Deep learning for echocardiography study diagram. **a** Two classification tasks were examined for echocardiography: view classification and left ventricular hypertrophy (LVH) classification. **b** Two different approaches for deep learning models were taken: a supervised pipeline model that performs segmentation (U-Net) before classification (CNN) and a semi-supervised generative adversarial network (GAN) for end-to-end learning. **c** The data, for both view and LVH classification, was split accordingly by study and no test data was utilized in training or validating the model

Previously,^19^ we explored supervised learning techniques for effective classification of echocardiogram view orientations, reporting a single image classification accuracy of 91.7% over 15 view classes. View classification is an important initial step in terms of a proof-of-concept to gain confidence that deep learning networks are performing accurately by learning relevant visual features, on-its-own as an assistant to sonographers for quality assurance, and as a precursor toward prediction of clinical diagnoses; as if an algorithm can learn what anatomical structure it is looking at, it can conceivably then learn patterns in what is defined as normal vs abnormal. We expand the previous work^19^ by examining both supervised and semi-supervised techniques for classification of view orientations, segmentation of relevant structures in echocardiogram images, and classification of left ventricular hypertrophy with the goal of automating cardiovascular disease prediction as captured in Fig. 1. Our expansion of work provides an expansion of the depth and breadth of both algorithms and applications in deep learning for echocardiography and medical imaging as a whole. We train convolutional neural networks (CNNs) on input resolutions of varying sizes and found an optimal balance between classifier performance and computational burden at 120 × 160 pixels. Performance of CNNs trained with default weight initialization were compared with transfer learning from Resnet50^20^ and VGG16,^21^ models that were pre-trained on the ImageNet^22,23^ dataset.

We report an accuracy of 94.4% on the same test set, using an ensemble of CNNs with a single U-Net^24^ for field of view segmentation. We applied the same techniques on a limited training dataset for single image LVH classification, obtaining a test accuracy of 91.2%. We show that generative adversarial networks (GANs),^25^ adapted for semi-supervised learning, can achieve better results than conventional CNNs in settings where labeled data is limited, achieving a test accuracy of 92.3% for LVH classification. Together, we present a working CNN system capable of accurately classifying left ventricular hypertrophy from a single echocardiogram image and a GAN system for automating disease predictions in data-limited settings.

## Results

### An optimum balance exists between resolution size and computational burden

**Fig. 2:**
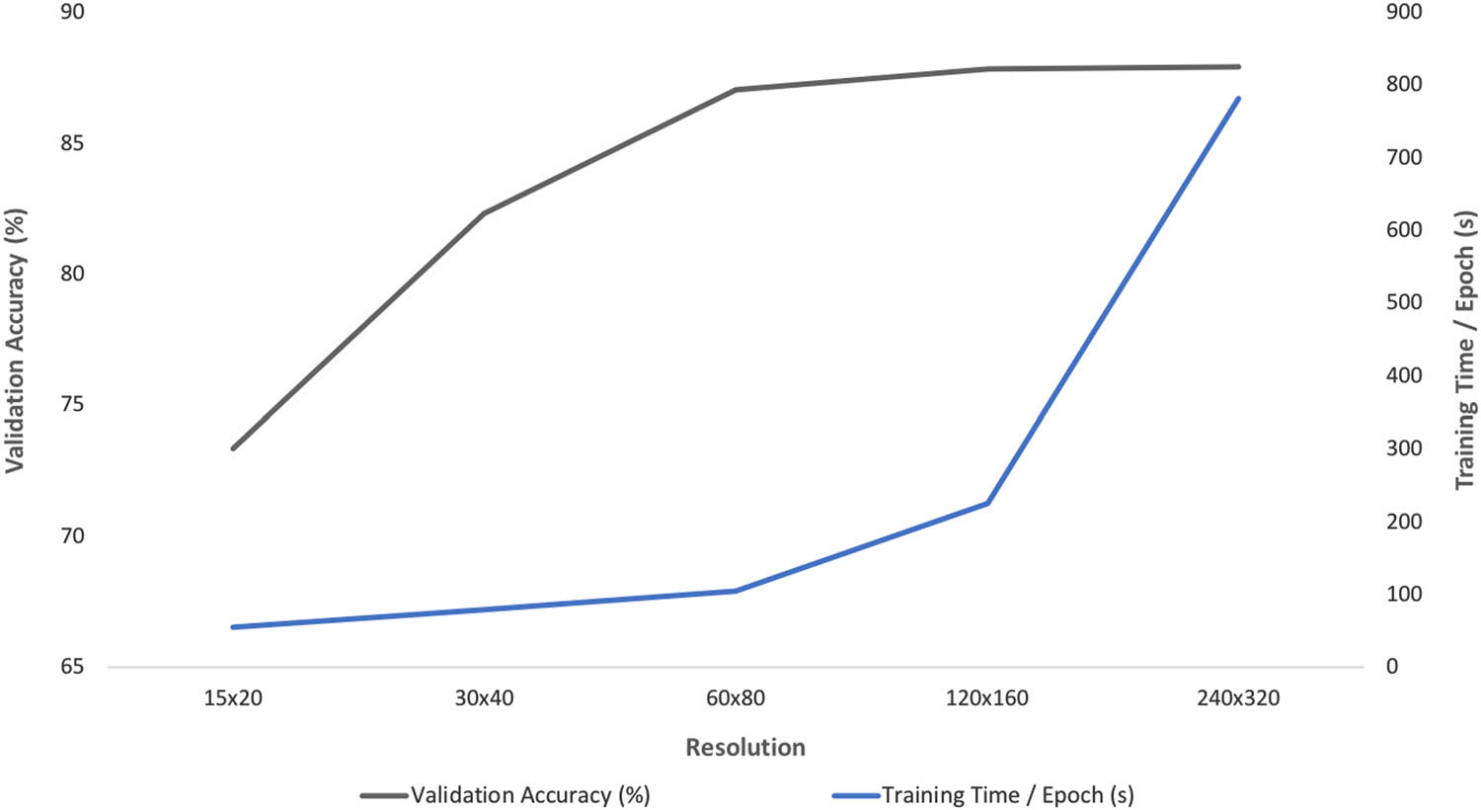
An optimal resolution size exists considering performance vs computational time tradeoffs. Plot of validation accuracy and training time per epoch with input resolution. The optimal balance between computational burden and validation accuracy exists at 120 × 160 resolution

We examined the effect of resolution size on accuracy performance and computational time. Ideally, there is an optimal point where the resolution is efficiently small without losing excessive structural information. For a subset of the echocardiographic data, we observed that with our VGG16-like architecture, validation accuracy plateaus at sub-88% accuracy for resolution above 60 × 80. As shown in Fig. 2 and Table 1, computation time scales with increasing input resolution. For convolution layers, we observed that filter sizes of 3 × 3, same padding, stride of 1 pixel, and pooling size of 2 × 2 works well generally across all resolutions, while maximum pooling works better than average pooling layers for resolutions above 60 × 80 pixels.

**Table 1:** Table of evaluation results for resolution study

Resolution	Training Accuracy	Validation Accuracy	Training Time / Epoch
240 × 320	94.49%	87.92% (+/−0.06)	781 s
120 × 160	98.43%	87.82 (+/−0.02)	226 s
60 × 80	99.10%	87.04% (+/−0.05)	104 s
30 × 40	93.57%	82.30% (+/−0.01)	79 s

Validation accuracy plateaus at sub-88% accuracy while training and testing time continue to scale with increasing input resolution.

### With only a few samples, custom deep learning segmenters can be trained to identify focus areas

**Fig. 3:**
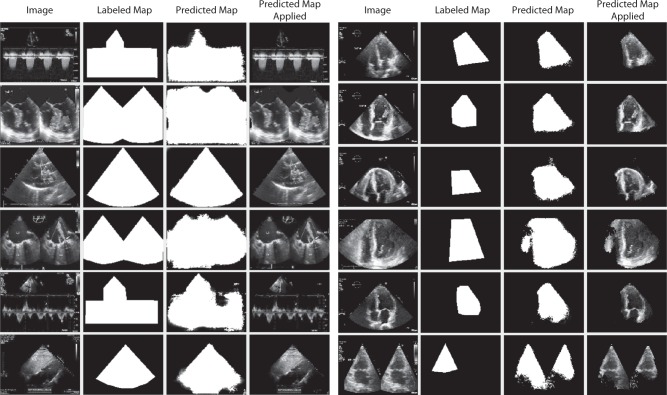
Figure of image data, labeled map, predicted map and predicted map applied to original image for Field of View (left) and LVH Segmentation (right). Trained segmentation models are able to accurately discern contours in echocardiogram images and output a map over relevant areas

Learning segmentation maps allows for focusing only the relevant pixels for subsequent classification. Our trained segmentation models are able to achieve satisfactory results with minimal labeling. The segmentation results for both Field of View and LVH segmentation are shown in Fig. 3. For View Segmentation, our model reported a pixel-wise cross-entropy loss of 0.3984 on the test set of 32 images. From visually inspecting the segmentation on our test set, segmentation of single mode images matches very closely to the labeled map, while segmentation of volumetric and dual mode is less consistent.

For Left Ventricle Segmentation, our model reported a pixel-wise cross-entropy loss of 0.1926 on the test set of 50 images. From visually inspecting the segmentation on our test set, segmentation of left ventricle is less tight compared to the labeled segmentation maps. Segmentation of images in dual mode is less consistent. While images in dual mode were labeled with a single mask around the left view, the model predicted segmentation maps around the left ventricle in both views.

### Field of view segmentation before classification achieves the highest reported accuracy for echocardiographic view classification

**Fig. 4:**
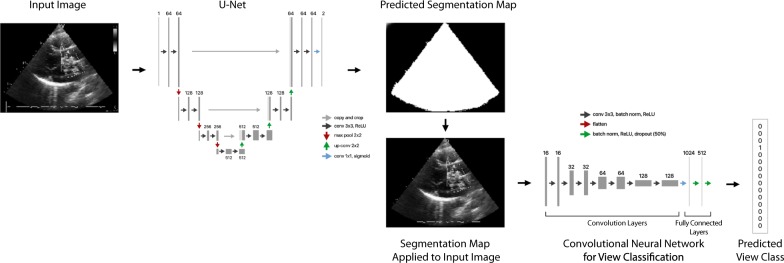
Field of View (FoV) segmentation and View Classification pipeline. Unet predicts a segmentation map over the main FoV, which is applied to the input image prior to view classification. FoV segmentation before view classification improved performance of the CNN model from an overall test accuracy of 92.05 to 93.64%

**Fig. 5:**
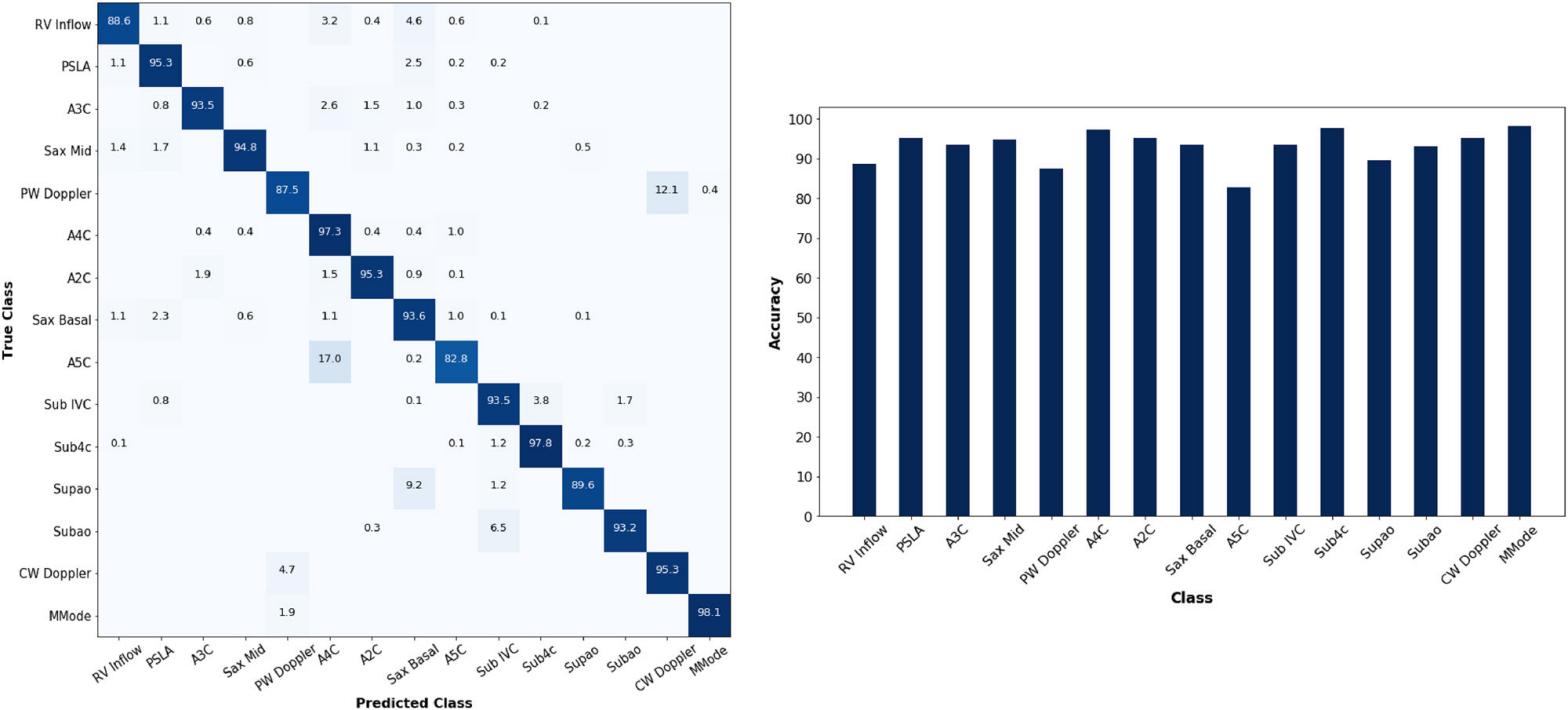
Normalized Confusion Matrix (left) and Accuracy by Class (right) on test set for ensemble network. 11 out of 15 classes achieved test accuracy above 90%. Classes with highest rate of confusion such as A5C with A4C are consistent with structural similarity overlap between the two classes

For echocardiographic 15-view classification, our CNN model without segmentation achieved an overall test accuracy of 92.05%. With the field of view segmentation prior to view classification pipeline as shown in Fig. 4, the same network architecture achieved an overall test accuracy of 93.64%. We found that average pooling layer outperforms maximum pooling layers for input images with Field of View segmentation. The best-performing model in our experiment is an ensemble of 3 CNNs with Segmentation that achieved an overall test accuracy of 94.40%. The resulting performance of each model is tabulated in Table 2. The normalized confusion matrix and accuracy by individual class for the ensemble is reported in Fig. 5. Both Resnet50 and VGG16 models achieved lower overall test accuracy of 91.36 and 83.67% respectively, despite having a more complex and deeper architecture.

**Table 2:** Table of evaluation results for each model for view classification

Model	Train Accuracy	Test Accuracy	Training Time/Epoch
CNN	95.39%	92.05% (+/−0.72)	919 s
CNN with Segmentation	95.18%	93.64% (+/−0.61)	903 s
Resnet50*	92.61%	91.36% (+/−1.43)	7482 s
VGG16*	98.15%	83.67% (+/−2.68)	4559 s
			(second stage)
Ensemble	—	94.40% (+/−0.52)	—

Highest performing model by overall test accuracy is an ensemble of three CNN models with field of view segmentation, while transfer learning from Resnet50 and VGG16 models yielded lower test accuracies compared to a single CNN model.

As segmentation is performed in the preprocessing step, test and training time corresponds with the size of the network architecture, transfer learning with Resnet50 took more than 8 times the amount of time per epoch compared to the CNN models.

### Left ventricle segmentation and transfer learning enables efficient classification of LVH using convolutional neural networks

**Fig. 6:**
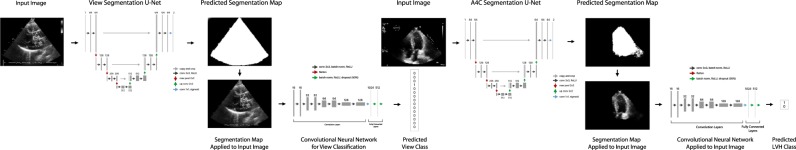
View classification and left ventricular hypertrophy classification pipeline. View segmentation Unet predicts a segmentation map over the main FoV, which is applied to the input image prior to view classification. Based on the predicted view class, input image is routed to the respective disease classification pipeline. a4c images are routed to a4c segmentation Unet, which predicts a segmentation map over the left ventricle. This map is applied to the input image prior to LVH disease classification

**Fig. 7:**
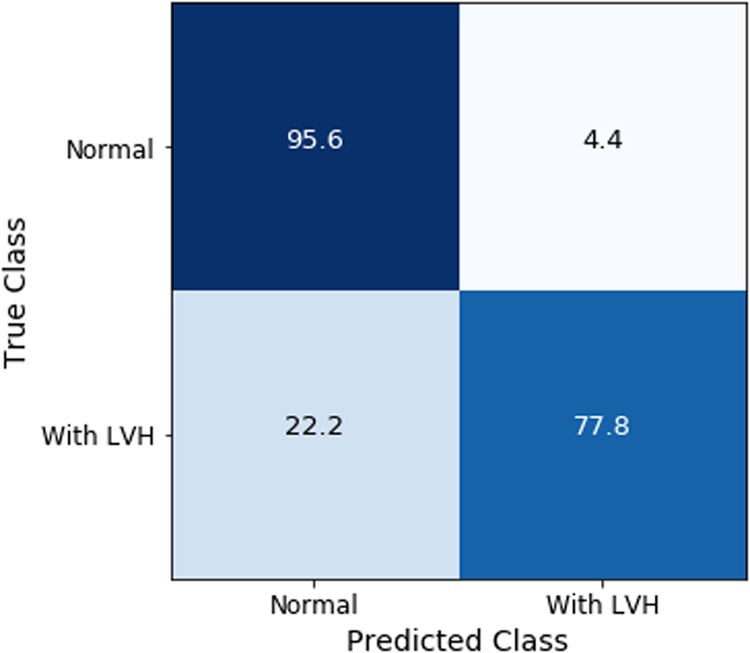
Normalized confusion matrix on LVH test set for CNN with segmentation stage 2. CNN model with segmentation was able to classify test images with 91.21% (+/−0.41) accuracy (specificity 95.70%, sensitivity 76.70%)

For left ventricle hypertrophy classification, a pipeline model of segmenters and classifiers was developed as shown in Fig. 6. As shown in Table 3, after the first stage training our convolutional neural network model with image segmentation pipeline achieved an overall test accuracy of 81.318% and F1 Score of 0.5952 on our test set of 182 images. We evaluated the model after the second stage training, and an overall test accuracy of 91.208% and an F1 Score of 0.8139 were measured. The normalized confusion matrix is reported in Fig. 7. CNN with segmentation outperformed our CNN network on both test accuracy and F1 score. In addition, we attempted training the CNN network with default initialization but the network failed to learn well and accuracy was very low.

**Table 3:** Test accuracies and F1 Scores for LVH classification models

Model	Test Accuracy	F1 Score
CNN (Stage 1)	84.07% (+/−1.23)	0.6027
CNN (Stage 2)	87.91% (+/−0.57)	0.7381
CNN with Segmentation (Stage 1)	81.32% (+/−0.98)	0.5952
CNN with Segmentation (Stage 2)	91.21% (+/−0.41)	0.8139

In stage 1, weights for convolution layers were fixed and model was trained over 20 epochs. In stage 2, weights of fully-connected layers were fixed and convolution layers were fine-tuned.

### Semi-supervised generative adversarial networks enable even higher classification accuracy for scenarios with large sets of unlabeled data

**Fig. 8:**
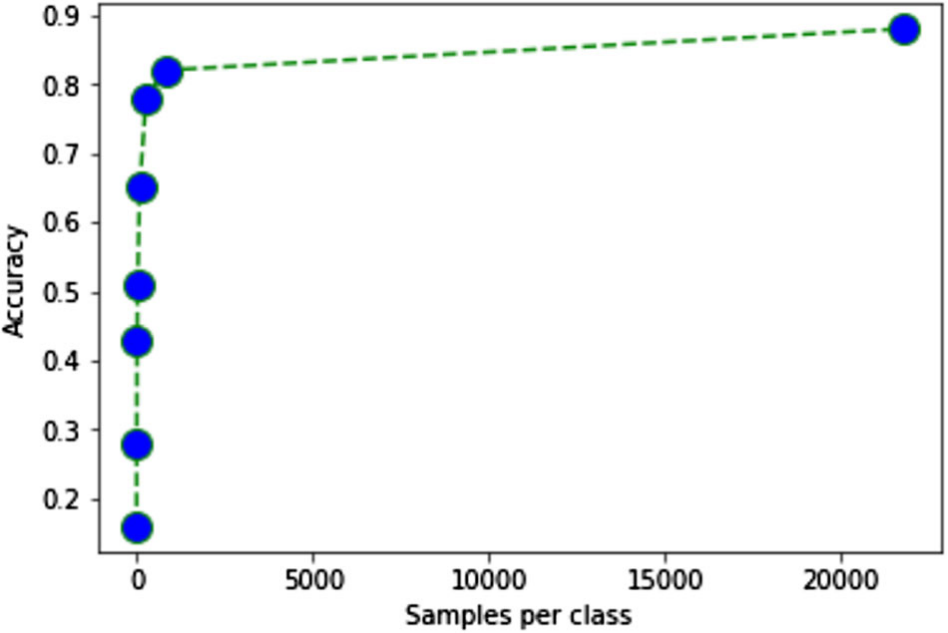
View classification performance of the semi-supervised generative adversarial network for varying amounts of labels as input. The model is able to learn from very small amounts of labeled data (approximately 4% of labels kept with the remaining data as unlabeled) to achieve greater than 80% accuracy for view classification. There exists an exponentially asymptotic behavior over number of labeled samples where accuracy gain becomes less prominent

The advantage of semi-supervised GANs is the utilization of all available data, whether labeled or unlabeled. For the view classification task, we studied the effect of training on varying amounts of labeled data by artificially apportioning part of the data as labeled and the remaining as unlabeled. The accuracy on the same test set for the various training scenarios is presented in Fig. 8 and Table 4. As observed, the relationship between increasing number of labels and model accuracy is highly exponential. With less than 4% of the data, the model is able to achieve greater than 80% accuracy.

**Table 4:** View classification performance of the semi-supervised generative adversarial network for varying amounts of labels as input

Labels per class	Test Accuracy
1	16%
5	28%
15	43%
30	51%
90	65%
270	78%
810	82%
21732	88%

**Fig. 9:**
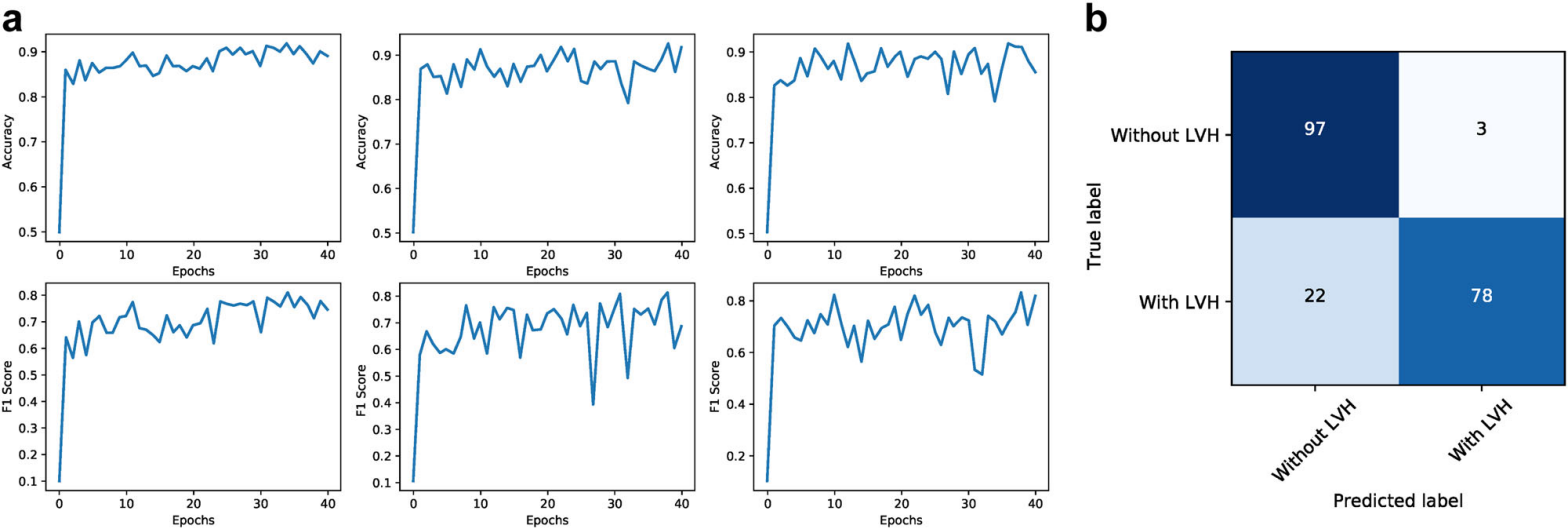
Performance of semi-supervised GAN for LVH classification in apical 4 chamber echocardiogram images. **a** For three separate models trained, the accuracy (top row) and F1-score (bottom row) is plotted vs number of epochs. The model training reliably reaches convergence and continues to fluctuate within a reasonable limit. **b** Normalized confusion matrix for an ensemble of the three models. This achieves an F1-score of 0.83 and accuracy of 92.3% (+/−0.57)

For the LVH classification task, we have the scenario of a small labeled data set (~2000 samples) but access to a large unlabeled data set (~76000). We assume the distribution of LVH in the unlabeled data is as probably more severely unbalanced than the labeled data, yet it does not affect our training methodology. In Fig. 9, we trained three separate models on the LVH dataset and compared the accuracies and F1 scores. We also plot the confusion matrix for an ensemble model comprising the three models that were trained on the LVH dataset. The accuracy rates and F1 scores for this task are higher for semi-supervised GANs than for the pipeline model technique described above.

**Fig. 10:**
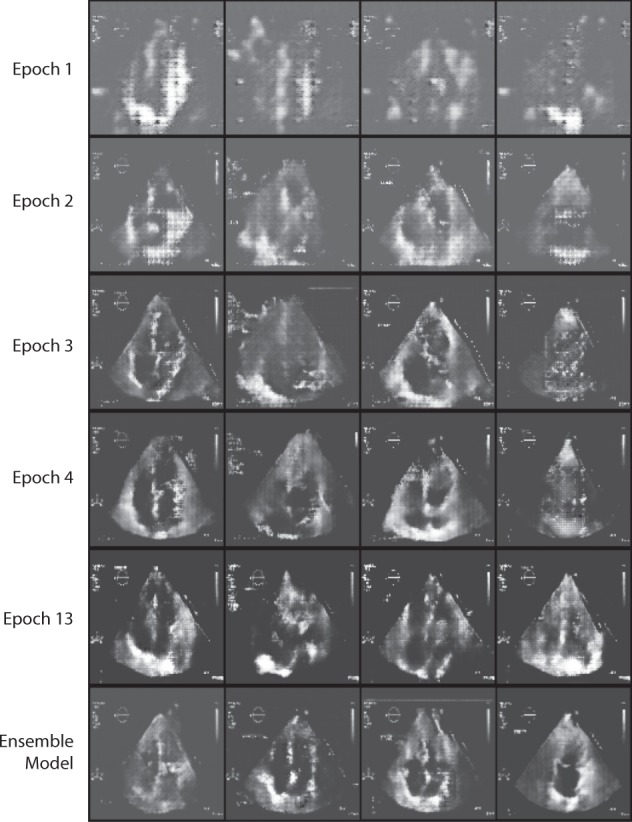
Generated images sampled from the generator network of the semi-supervised GAN during training for LVH classification. For one model, batches of size four are shown containing generated images (top to bottom) after epoch 1, 2, 3, 4, 13. The last row displays generated images from an ensemble of three GAN models. Qualitatively, the model clearly learns and understands the underlying physiological structures in input distribution

Figure 10 shows the progression of results from the generator which was used in the GAN training process to perform semi-supervised learning. We observe the quality of generated samples improves after a few epochs and that medically-relevant structures are discernable by the time of convergence as opposed to random noise. This affirms that the model is capturing and understanding the relevant features that comprise our data distribution.

## Discussion

Deep learning has revolutionized the development of automated algorithms across multiple computer vision tasks.^5^ For each domain, there are specific considerations that become more relevant in the development of deep learning models. In particular, medical imaging—across modalities—will often come in a DICOM format and have either high resolution images or varying resolution images per acquisition. Pre-processing the data to standardize the resolution is a prerequisite step before building deep learning models with non-trivial implications downstream. Keeping all pixel information can be superfluous and lead to excessive number of weights to learn and computation time. Downsampling is usually performed on input images while attempting to avoid loss of visual features that can be used to distinguish between multiple classes. Our results show that for a computer vision domain and task, we can quantify a study of ideal resolution size. For our study, there exists a critical resolution at around 60 × 80 pixels which provides the minimal amount of visual information necessary for accurate view classification. Naturally, the ideal amount of downsampling will vary based on the size and complexity of visual structures for a particular task. However in general, the computation time increases rapidly with increasing input resolution as the network architecture increases in depth and convolution per layer quadruples. We also found that the model architecture with highest validation accuracy varies across each resolution. For 240 × 320 resolution, a filter size of 7 for first two convolution layers outperforms a filter size of 3 as a 3 × 3 patch of the input image is contains insufficient visual information for effective classification. In the varying resolution study, our training set had less than a quarter of the available training data to allow for rapid experimentation. While we attempted to identify the best architecture for each resolution, there remains scope for further optimization.

Our segmentation model effectively removes visual features that are less relevant for View and LVH classification. Utilizing segmentation before classification provides an elegant method to localize the attention of predictive models to pixels with relevant visual features. This is enabled and made practically feasible by the fact that only a small sample of labeled segmentation masks are required for training. These masking subtasks can be abstracted away to lower-cost labeling labor as well. Lastly, we observed that even in cases where there is a large difference between the labeled and the predict map, there is very limited amount of information loss after the predicted map is applied as shown in Fig. S2. The U-Net tends not to mask over areas where it is less confident of.

There is an improvement (+1.59%) in overall test accuracy with the segmentation pipeline compared to the initial CNN model, which confirms our initial hypothesis that the removal of auxiliary information using the U-Net segmentation model helps to simplify the view classification problem. While end-to-end learning is growing in popularity with the increasing size of datasets and network depth, having a sequential pipeline of different neural network models each performing a heuristic-based classification task that simplifies the classification problem for the next network can be more effective depending on the problem domain. We view this as the primary reason behind the improvement of view classification accuracy from our previous study.^19^

Transfer learning from pre-trained VGG16 and Resnet50 models was computationally expensive and failed to match the accuracy of the simpler CNN model with default weight initialization. While transfer learning has been used successfully in the domain of disease classification,^26^ this strengthens our view that transfer learning from models pre-trained on the ImageNet dataset may not be as effective or computationally feasible for datasets that with significant structural differences. Lastly, as with previously published results,^27^ we were able to exact accuracy improvements on our test dataset by applying an ensemble technique to average predictions of multiple models.

In our left ventricular hypertrophy study, we observed that using transfer learning techniques from the view classification model enables the neural network to learn more effectively compared to default weight initialization. Applying left ventricle segmentation to the apical 4 chamber images produced an even larger improvement in overall test accuracy compared to our view classification study, corresponding to the larger area of image removed during the segmentation step.

While the final model achieved 91.2% (+/−0.41) accuracy with 95.7% specificity and 76.7% sensitivity, it is within reasonable limits given that there were 4 times the amount of training data for the former. The success of our LVH classification model demonstrates that deep learning models adapted for echocardiogram datasets can generalize well from view classification to disease classification even with a small training set.

In addition to a pipeline deep learning model with solely supervised classifiers, semi-supervised GAN models were explored as a generalizable approach that leverages learning from both labeled and unlabeled data. For computer vision tasks in medical imaging, we often have scenarios where there is a larger unlabeled dataset and only a portion of the data can be accurately and cost-effectively ground-truthed.

The semi-supervised GAN model was trained and tested on the view classification problem first as we could designate varying proportions of data for labeled vs unlabeled to observe the effect on classification performance. The results show that semi-supervised GANs require an order of magnitude less labeled data (~4% of total data) to achieve adequate performance. We also saw that the GAN still performs well in asymmetrically distributed categories for our view classification. These results provided motivation to train for the LVH classification task where only a small portion of data existed with LVH labels (~2200 samples). For the LVH classification task, the semi-supervised GAN is able to learn from both the labeled and unlabeled data, even with highly unbalanced classes, to achieve an accuracy of 92.3% (+/−0.57), with specificity of 97.0% and sensitivity of 79.1%.

The semi-supervised GAN loss function is formulated to account for contributions from unlabeled, labeled, and generated samples. On a high level, the GAN through the sigmoid real/fake loss for unlabeled samples becomes better at training filters which can identify salient features of an image while the labeled samples are utilized mostly for implicitly training the layers which performs the final classification. GANs also implicitly are performing data augmentation as the generator converges—producing more realistic images which allow the GAN to explore probability spaces of the data not covered in the original training data. Future areas of exploration include more robust loss functions, conditioning the generated noise, and normalization techniques.^28,29^

One of the most important aspects of our study is the usage of GANs for biomedical image classification tasks. Although the particular hyper-parameters and implementation details may vary outside this dataset and prediction task, the overall model considerations in addition to the data and experimental pipeline is applicable across medical domains and tasks. We shed light on the development and improvements of semi-supervised GANs, relevant performance metrics, and functional intuitions as to promote its application both within and beyond echocardiography and cardiology. To do so, we experiment with two different prediction tasks in addition to a comparative analysis with reference to traditional deep learning architectures such as a CNN.

As with all studies, there are natural limitations to our work. It is important to be stated that although we focus on improving image classification tasks in echocardiography, image classification is only one aspect of clinical diagnosis as clinicians utilize other forms of cognition to both understand and treat a patient’s illness. We view this line of work as not a replacement of the clinician but as an assistant for relevant tasks that a clinician performs. Also, it is also worth noting that our sample sizes are limited due to practical reasons—which is a common medical imaging issue that beckons for data-efficient, generalizable techniques presented in this study. We look forward to future work and validation that is conducted on even larger sample sizes that include both more patients and also variations in acquisition (i.e. different institutions, field of views, and more).

To conclude, the focus of our study is on data-efficient deep learning models for classification tasks in medical imaging. Initially, we investigate the trade-off between resolution size and computational burden. We then explore two main techniques: (1) supervised pipeline models to first extract relevant structures then pass through a CNN classifier (2) semi-supervised GAN models for end-to-end training. With customized segmentation models and the extra effort to label additional segmentation maps, the first method is able to achieve the highest reported accuracy for view classification in cardiac ultrasound imaging and relatively high performance for LVH classification. Often in practice, labeled data in medical imaging is scarce, locked by privacy or regulatory concerns, or expensive to annotate. Utilizing both labeled and unlabeled data and with a generalizable end-to-end training strategy, the second method is able to achieve high performance for LVH classification. We provide avenues and trade-offs for practitioners and researchers with the aforementioned techniques for their computer vision tasks in medical imaging.

## Methods

### Echocardiographic Data

All datasets were obtained and de-identified, with waived consent in compliance with the Institutional Review Board (IRB) at the University of California, San Francisco (UCSF). Methods were performed in accordance with relevant regulations and guidelines. Echocardiographic studies were extracted then videos and still images were stripped of identifying metadata and flattened to individual frames. Data comprised of studies between 2000 and 2017 at random from UCSF’s clinical database. These studies included men and women (49.4 and 50.6 percent, respectively) ages 20–96 (median age, 56; mode, 63) with a range of body types (25.8 percent obese) and were acquired with equipment from several manufacturers (eg. GE, Philips, Siemens). For view classification, the number of studies taken were N = 267 and fifteen common views were utilized including: parasternal long axis, right ventricular inflow, basal short axis (aortic valve level), short axis at mid (papillary muscle) or mitral level, apical two chamber, apical three chamber (apical long axis), apical four-chamber, apical five chamber, subcostal four-chamber, subcostal inferior vena cava (IVC), subcostal abdominal aorta, suprasternal aortic arch, pulsed-wave Doppler, continuous-wave Doppler, and m-mode. For LVH classification, the number of studies taken were N = 455 and the diastolic frames in the apical 4 chamber view were selected and comprised of the following clinical labels: normal, moderate-to-severe asymmetric LVH, severe asymmetric LVH, moderate concentric LVH, moderate-to-severe concentric LVH, severe LVH. Further information regarding source data can be found in the previous literature.^19^

### Varying Resolution Study

**Fig. 11:**
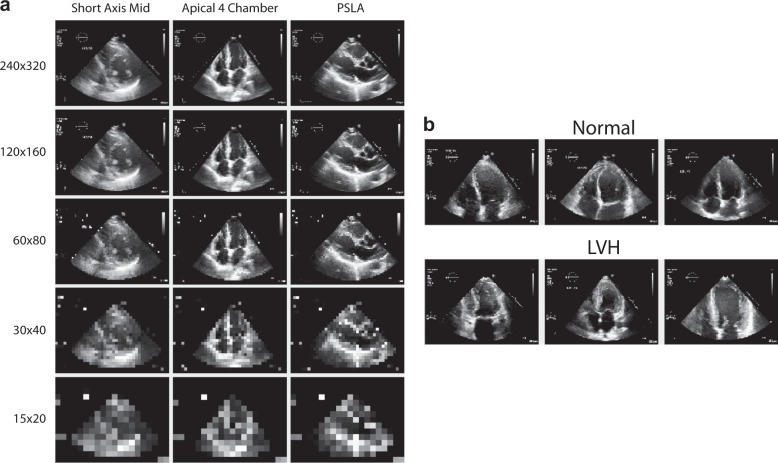
Sample data from echocardiographic studies. **a** Sample echocardiogram images at varying resolutions (rows) for three example views (columns). Selection of the optimal resolution is influenced by the tradeoff between classifier performance and computational time. **b** Sample apical four chamber echocardiography images with and without Left Ventricular Hypertrophy (LVH). LVH is characterized by the thickening of the left ventricle—a perilous condition increasing the risk of myocardial infarction, stroke, and death

We divided our subset of 103102 images into a training set containing 75937 images, a validation set containing 11696 images and a test set containing 15469 images, in an approximate (74:11:15) split. There is no patient overlap between the training, validation and test set. Downsizing of images originally in 600 × 800 pixels to the image resolutions of 240 × 320, 120 × 160 60 × 80, 30 × 40 and 15 × 20 pixels were completed using Scipy’s imresize function with the nearest interpolation mode. Figure 11a displays sample images from each resolution for three classes.

Our neural network architectures were designed in Python using the Keras library with TensorFlow backend,^30^ primarily based on the VGG16 Network,^21^ which won the ImageNet Challenge in 2014.^22,23^ Our architecture consists of multiple convolution layers, followed by fully connected layers. We applied Batch Normalization^31^ and rectified linear activations^32^ after each layer. Softmax activation was applied to the final fully connected layer for classification over the 15 output classes. Dropout^4^ and L2 Regularization were added in the fully-connected layers to prevent overfitting.

We further experimented with various architectures for each resolution and the architectures with the highest validation accuracy are reported in the Supplementary Material. For convolution layers, filter sizes of 3 × 3, same padding, stride of 1 pixel, and pooling size of 2 × 2 were used for each resolution except 240 × 320, for which a filter size of 7 × 7 were used for the first two convolution layers. Maximum Pooling were used for resolutions 60 × 80 pixels and above, and Average Pooling for resolutions below 60 × 80 pixels.

Data augmentation was applied during training with up to 10 degrees rotation and 10% height and weight shifts. Adam optimizer with default parameters was used to minimize the categorical cross-entropy loss. Learning rate was set to 0.02, with decay per epoch of 0.85. Early stopping was applied once validation loss stops decreasing for two consecutive epochs. Training was performed on Nvidia GTX1080Ti GPUs, with batch sizes of varying sizes used for the different resolutions to maximize GPU memory usage. We evaluated our final models by computing the overall validation accuracy, average time to train and validate per epoch.

### Relevant Structure Segmentation

From our varying resolution study, we selected 120 × 160 as the optimal resolution for our subsequent experiments. It provided an ideal balance between accuracy and computational time as discussed in Results.

We employed relevant structure segmentation as preprocessing for removal of irrelevant details in the images to simplify the classification task. We trained a convolutional neural network on two different datasets for segmentation of the main field of view (FoV) in echocardiogram images prior to view classification, and segmentation of the left ventricle in apical 4 chamber (a4c) images prior to left ventricular hypertrophy classification.

Our field of view segmentation dataset contains 433 images- inclusive of image frames from various 2D views, doppler, and m-mode echocardiograms. Images were converted to grayscale and downsampled to 120 × 160 pixels using Scipy’s imresize function with the nearest interpolation mode. The data were divided by class into a training set consisting of 411 images and a test set consisting of 32 images for evaluation of the model’s performance.

Masks were drawn on the outline of the field of view containing the medical image with an in-house labeling tool that the user selects vertex points to form a polygon shape. For doppler and m-mode echocardiograms, masks were drawn around the general shape of the waveforms and field of view. The labeling tool is an interactive polygon editor built with matplotlib that sets pixels inside the mask to 1 and pixels outside to 0.

Our left ventricle segmentation dataset contains 720 images sampled randomly from the apical 4 chamber (a4c) view dataset. Some apical 4 chamber views might be cropped and the left ventricle may not be visible. Therefore, for Relevant Structure Segmentation for LVH only, we did not include images that failed to show the left ventricle. Important to emphasize, no filtering was performed for the final LVH test set images or calculation of performance metrics on the final goal of LVH classification. Images were downsampled to 120 × 160 pixels using Scipy’s imresize function with the nearest interpolation mode. Images were divided into a training set consisting of 670 images and a test set consisting of 50 images. Using the aforementioned labeling tool, masks were drawn to create an approximate polygon around the left ventricle epicardium. Masks were drawn around the left ventricle using the labeling tool. For images in dual mode, a single mask was drawn around the left ventricle in the left-most view.

**Fig. 12:**
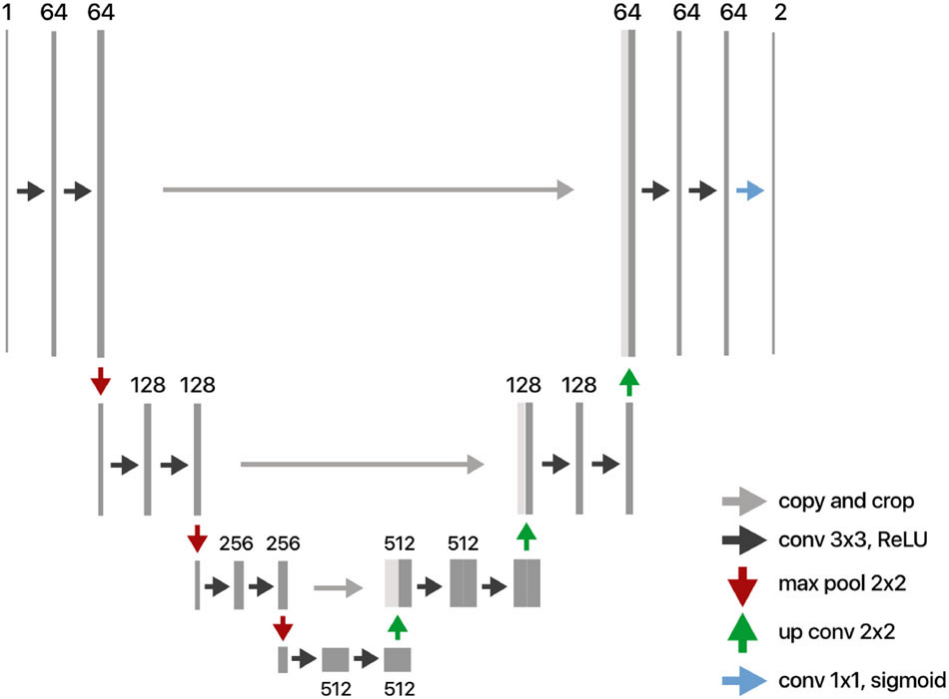
Modified U-net architecture used for segmenting relevant visual structures. The architecture consists of a contracting path and a symmetric expanding path—combining high resolution features from the contracting path and upsampled output for precise localization. Pixel-wise softmax is applied on the final output to produce a segmentation map

Our image segmentation model is based on the U-Net architecture^24^ and is shown in the Fig. 12.

Modifications were made to the original architecture for adaptation to the 120 × 160 pixels resolution of our dataset. These include changes to filter sizes, removal of convolution layers with 1024 filters and the addition of Dropout before the first up-sampling convolution layer.

The model was trained over 50 epochs with a learning rate of 0.0001 and per epoch decay of 0.93 using the Adam optimizer.

We evaluated the model by computing the pixel-wise loss on the test set. We also visually inspected the U-Net’s segmentation on the test set to further verify the segmentation performance.

### Echocardiographic View Classification

Our dataset consists of 347726 echocardiogram images, of which 325980 images were in the training set. The original test set used in the previous study^19^ containing 21746 images were retained for this experiment.

Images were downsampled to 120 × 160 pixels using Scipy’s imresize function with the nearest interpolation mode for the convolutional neural network (CNN) experiments. Images used for Resnet50 and VGG16 were resized and copied over all three channels to fit the pre-trained models input dimensions of 224 × 224 × 3.

In this experiment, we trained the following models to compare the effectiveness of relevant structure segmentation for preprocessing, transfer learning and ensembling: CNN model trained using the original images, CNN model trained using images with FoV Masking, Transfer Learning from Resnet50 and VGG16 models using images with FoV Masking and an Ensemble of 3 CNN models trained using images with FoV Masking.

We retained the same architecture for 120 × 160 resolution as used in the varying resolution study, with the exception of replacing Max Pooling layers with Average Pooling layers for the CNN trained with FoV Masking.

Resnet50 and VGG16 models from the Keras Library which were pre-trained on the ImageNet dataset were used. The fully-connected layers from the Resnet50 model were removed and replaced with a batch normalization layer followed by a fully connected layer with softmax activation. For VGG16, we replaced the fully-connected layers with the same fully-connected layers layers with batch normalization and L2 regularization, followed by a fully connected layer with softmax activation.

Our ensemble consists of 3 CNN models trained individually using images with FoV Masking, with the predictions output averaged.

Adam optimization with default parameters and early stopping were used for the following experiments. During training of the CNN models, learning rate was set to 0.02 with decay per epoch of 0.85. Training data was augmented with up to 10 degrees rotation and 10% height and weight shifts.

For the pre-trained models, training data was augmented with up to 5 degrees rotation, 10% height and 15% weight shifts. Fine-tuning for Resnet50 was performed end-to-end with a learning rate of 0.01 and decay per epoch of 0.96.

For VGG16, training was divided into two stages. In the first stage, weights from the convolution layers were frozen and the fully connected layers with Xavier initialization were trained till convergence with a learning rate of 0.02 and decay per epoch of 0.9. In the second stage, weights from the convolution layers were fine-tuned along with the fully connected layers at a lower learning rate of 0.001 and decay of 0.9 per epoch.

We evaluated the various models by computing the overall test accuracy across 15 classes, training and test time per epoch, test accuracy by class, confusion matrix, and F1 score, which is the harmonic average of precision and recall. Confidence intervals were computed by the bootstrapping technique with replacement for 95% intervals.

### Left Ventricular Hypertrophy Classification

The dataset consists of 2269 images from the apical 4 chamber (a4c) view. The first two frames of an echocardiographic study were selected to ensure only diastolic phase. To formulate this as a binary classification problem as in Fig. 11b, we chose images with different labels for Left Ventricular Hypertrophy (LVH) to form a single class with 462 images. 1807 images of normal a4c views formed the other class. The ratio of images without LVH to images with LVH is approximately 4:1.

Image were divided into a training set consisting of 1890 images, validation set of 172 images and a test set of 207 images. Images from the same patient were placed in the same split. For preprocessing, images were downsampled to 120 × 160 pixels and masked with the predicted output from the left ventricle segmentation model in our previous experiment.

We retained the same architecture for 120 × 160 resolution as used in the varying resolution study, with the exception of replacing Max Pooling layers with Average Pooling layers. L2 regularization of 0.03 and Dropout of 0.4 was applied to the fully connected layers.

We applied a two-stage transfer learning method for the training of this model. Weights for convolution layers were initialized with weights from the CNN model trained using images with FoV Masking. Fully connected layers were initialized with Xavier uniform initialization.

In the first stage, weights of convolution layers were fixed and the model was trained over 20 epochs to minimize binary cross-entropy loss, with a learning rate of 0.025 and decay per epoch of 0.85. In the second stage, weights of fully-connected layers were fixed and convolution layers were fine-tuned with a learning rate of 0.001 and decay per epoch of 0.9. Early stopping was applied once validation loss stops decreasing for two consecutive epochs.

We evaluated the model by computing the test accuracy and F1 score, which is the harmonic average of the precision and recall. Confidence intervals were computed by the bootstrapping technique with replacement for 95% intervals.

### Semi-supervised Generative Adversarial Networks

**Fig. 13:**
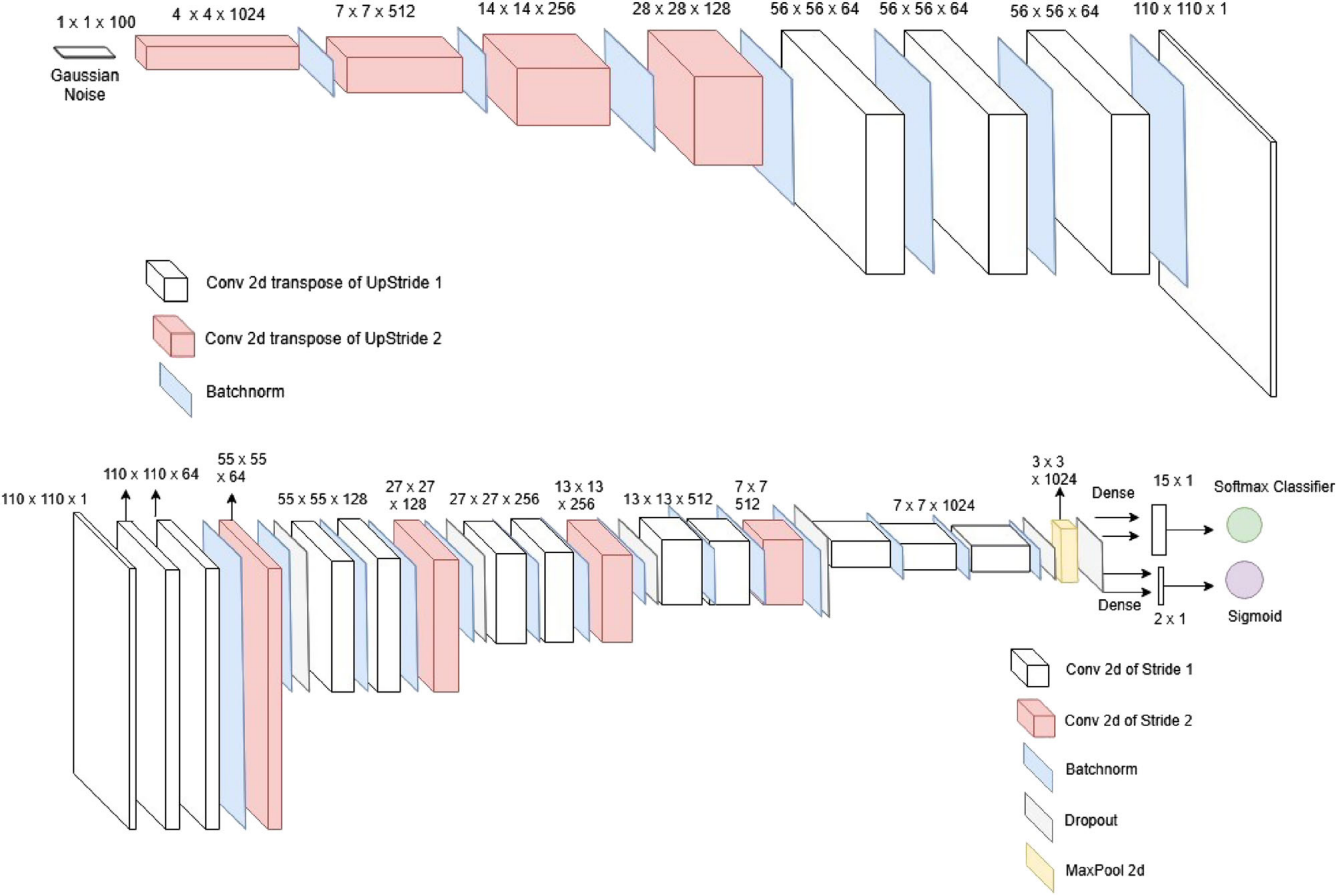
Semi-supervised GAN for echocardiogram view and LVH classification. Generator (top) consists of a Gaussian noise layer which gets passed through conv-transpose layers to output images of size 110 × 110. Discriminator (bottom) downsamples original images with regular strides of two every three layers, resulting in a softmax output for labeled (or supervised) loss and a sigmoid output for unsupervised loss

As shown in Fig. 13, the GAN makes use of two neural networks: a generator which attempts to generate realistic images and a discriminator which discriminates between real (optionally including specified classes) and fake.^25^The same semi-supervised GAN architecture is used for both the view classification task and the left ventricular hypertrophy classification task.

The discriminator model is structured in blocks of three convolutions comprising two convolutional layers of stride 1 and one convolutional layer of downstride 2. All except the last convolution layer make use of 3x3 convolutional filters, batch normalization layer after each convolution layer and dropout layer after every downstride convolution layer. Leaky RELUs are used as activation functions for all the layers and the last classification layers which use Softmax and Sigmoid.

The generator model consists of seven deconvolutional layers each of stride two and it works by progressively upsampling from the gaussian noise layer where the model begins. Each of these layers use either a 3x3 or 4x4 convolutional filter and a batch normalization layer. All intermediate activations consist of RELUs while the very last layer makes use of Tanh activation to output the final image.

Training GANs for semi-supervised learning involves performing three passes through the discriminator and one pass through the generator at each iteration. To compute the discriminator loss a labeled image is passed through the discriminator to assess a cross-entropy loss. Then an unlabeled image and a fake image are passed through the discriminator for both of which we compute binary cross entropy losses. All three of these losses are summed together and are used to perform a single step of backpropagation through the discriminator. The losses were inspired by previous literature^33^ and detailed below:

1 $$L = L_{supervised} + L_{unsupervised} + L_{generated}$$

2 $$L_{supervised} = - {\mathbf{E}}_{x,y\sim p_{data}(x,y)}{\mathrm{log}}\,p_{model}(y|x,y < K + 1)$$

3 $$L_{unsupervised} = - {\mathbf{E}}_{x\sim p_{data}\left( x \right)}{\mathrm{log}}\left[ {1 - p_{model}\left( {y = K + 1{\mathrm{|}}x} \right)} \right]$$

4 $$L_{generated} = - {\mathbf{E}}_{x\sim G}{\mathrm{log}}\left[ {p_{model}(y = K + 1|x)} \right.$$

To compute the generator loss, we generate a fake image from the generator which we pass through the discriminator and compute the loss by using a mean square error loss between the second last layer of the discriminator for our fake image and for an unlabeled image.

The data used for view classification was the same as the models outlined above, including the size of the splits. The only differences were that the image was downsized to 110 × 110 pixels and that the split between unlabeled data and labeled data was changed for different models so as to determine the relationship between labels and accuracy. One epoch was defined as the number of unlabeled images since this was always going to be the larger value. This however meant that in each epoch the discriminator ends up going through multiple epochs of the labeled images.

For LVH classification problem we used the same labeled data consisting of 2269 images which were divided into a training set consisting of 1915 images, validation set of 172 images and a test set of 182 images. Images from the same patient were placed in the same split. The only difference was that we also used 76404 unlabeled images (from the apical 4 chamber data in the view classification dataset) on top of the labeled ones to bolster the semi-supervised GAN. For preprocessing, these images were downsampled to 110 × 110 pixels.

Training was performed using the Adam optimizer set at default values for both of these tasks. A learning rate of 0.0003 with no decay mechanism. Training a GAN using 2 GTX 1080Ti GPUs training was on the order of five hours.

To evaluate the performance of semi-supervised GAN for view classification we only made use of the accuracy rate of the discriminator model at different epochs. To evaluate the performance of the LVH model we use accuracy rate of three models trained on all of the data as described above. We also use the F1 scores of these models and plot them on a separate curve. For the highest performing model, we used confusion matrices to better illustrate how the model performs on different categories.

### Code Availability Statement

The code developed in this study is available from mofrad@berkeley.edu on reasonable request.

## Electronic supplementary material

Supplemental Information

## Data Availability

The datasets utilized during this study are not publicly available due to reasonable privacy and security concerns. The data is not easily redistributable to researchers other than those engaged in the Institutional Review Board-approved research collaborations with the University of California, San Francisco (UCSF).

## Electronic supplementary material

**Supplementary information** accompanies the paper on the *npj Digital Medicine* website (10.1038/s41746-018-0065-x).
