# Supplementary material for: Deep echocardiography: data-efficient supervised and semi-supervised deep learning towards automated diagnosis of cardiac disease
Source: NPJ Digit Med. 2018 Oct 18;1:59. doi: 10.1038/s41746-018-0065-x (PMC6550282; doi:10.1038/s41746-018-0065-x)
Supplement: Supplementary file 1 — Supplemental Information [file 41746_2018_65_MOESM1_ESM.pdf]

Supplementary Material

240x320 Resolution

	# Filters	Filter Size	Dropout	L2
Conv	8	7	-	-
Conv/Maxpool	8	7	-	-
Conv	32	3	-	-
Conv/Maxpool	32	3	-	-
Conv	32	3	-	-
Conv/Maxpool	32	3	-	-
Conv	64	3	-	-
Conv/Maxpool	64	3	-	-
Conv	128	3	-	-
Conv/Maxpool	128	3	-	-
FC (1024 units)	-	-	0.5	0.02
FC (512 units)	-	-	0.5	0.02

120x160 Resolution

	# Filters	Filter Size	Dropout	L2
Conv	16	3	-	-
Conv/Maxpool	16	3	-	-
Conv	32	3	-	-
Conv/Maxpool	32	3	-	-
Conv	64	3	-	-
Conv/Maxpool	64	3	-	-
Conv	128	3	-	-
Conv/Maxpool	128	3	-	-
FC (1024 units)	-	-	0.5	0.02
FC (512 units)	-	-	0.5	0.02

60x80 Resolution

	# Filters	Filter Size	Dropout	L2
Conv	32	3	-	-

Conv/AveragePool	32	3	-	-
Conv	64	3	-	-
Conv/AveragePool	64	3	-	-
Conv	128	3	-	-
Conv/AveragePool	128	3	-	-
FC (1024 units)	-	-	0.5	0.02
FC (512 units)	-	-	0.5	0.02

30x40 Resolution

	# Filters	Filter Size	Dropout	L2
Conv	16	3	-	-
Conv/AveragePool	16	3	-	-
Conv	32	3	-	-
Conv/AveragePool	32	3	-	-
Conv	64	3	-	-
Conv/AveragePool	64	3	-	-
Conv	128	3	-	-
Conv/AveragePool	128	3	-	-
FC (1024 units)	-	-	0.6	0.02
FC (512 units)	-	-	0.6	0.02

15x20 Resolution

	# Filters	Filter Size	Dropout	L2
Conv	64	3	-	-
Conv/AveragePool	64	3	-	-
FC (1024 units)	-	-	0.4	0.02
FC (512 units)	-	-	0.4	0.02

Fig S1. Tables detailing architecture of CNNs used for Varying Resolution Study. Input resolution from top: 240x320, 120x160, 60x80, 30x40. Batch normalization and by ReLU applied after convolution (Conv) and fully connected (FC) layer. For each architecture, an additional fully connected layer with softmax activation outputs prediction over 15 classes. Convolution layers have same padding and stride of 1. Pooling size of 2x2 used for Average and Max Pooling layers.

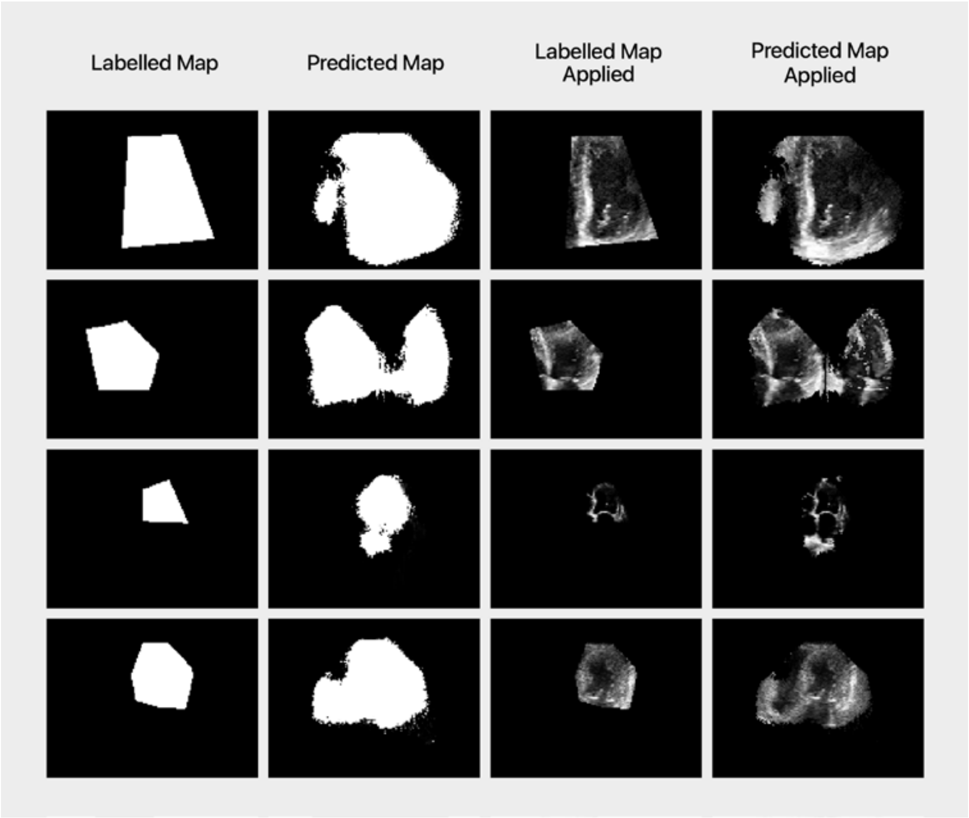

Fig S2. Figure of labeled map, predicted map, labeled and predicted map applied to original image for LVH Segmentation. Even in cases where there is a large difference between the labeled and the predict map, the amount of information loss after applying the predicted map is very limited.
